# Retail food environment around higher education institutions in a Brazilian metropolis

**DOI:** 10.1590/1980-549720250004

**Published:** 2025-01-31

**Authors:** Larissa Edwiges Ananda da Silva, Thales Philipe Rodrigues da Silva, Olívia Souza Honório, Monique Louise Cassimiro Inácio, Larissa Loures Mendes

**Affiliations:** I Universidade Federal de Minas Gerais, Postgraduate Program in Nutrition and Health - Belo Horizonte (MG), Brazil.; II Universidade Federal de Minas Gerais, Postgraduate Program in Nursing - Belo Horizonte (MG), Brazil.; III Universidade Federal de São Paulo, Paulista School of Nursing, Department of Nursing in Women's Health - São Paulo (SP), Brazil.; IV Universidade Federal de Ouro Preto, Postgraduate Program in Health and Nutrition - Ouro Preto (MG), Brazil.; V Universidade Federal de Minas Gerais, Department of Nutrition - Belo Horizonte (MG), Brazil.

**Keywords:** Built environment, Universities, Young adult, Geographic mapping, Public health

## Abstract

**Objective:**

To analyze the retail food environment and identify the presence of food swamps around public and private higher education institutions (HEIs) in Belo Horizonte, Minas Gerais.

**Methods:**

This is an ecological study with the analysis unit being a 500-meter buffer network around 81 in-person HEI units. The density and proximity between the HEIs and food purchasing establishments for immediate consumption were assessed according to the administrative category and *per capita* income of the census tract, as well as the presence of food swamps.

**Results:**

In 98.76% of the buffers there was at least one establishment for immediate consumption. Snack bars, restaurants, and bars were the categories most available and closest to the HEIs. The density of establishments was higher around private HEIs and around HEIs located in higher income areas. It was found that 95.06% of HEIs were located in areas classified as food swamps.

**Conclusion:**

Thus, the HEIs evaluated were exposed to neighborhoods with an unhealthy food environment, which may predispose university students to food choices based on the consumption of ultra-processed foods and alcoholic beverages.

## INTRODUCTION

The organizational food environment of universities, often referred to as the university food environment (UFE), is defined by elements of the institution's internal physical environment and its surrounding areas, which are considered integral components^1^. According to Castro et al.^1^, the surrounding areas encompass the physical and "virtual" contexts associated with food options available to individuals frequenting a given organizational environment but not directly influenced by the organization's management. The physical context includes establishments selling food, beverages, and culinary preparations, as well as informal trade of such products in the areas adjacent to the organization.

University students often bring meals from home or purchase food off campus^2^, indicating that the influence of UFE on eating habits extends beyond what is available within higher education institutions (HEIs). Additionally, the university population, primarily composed of young adults, has the autonomy to acquire food in areas surrounding the institution, as there are no restrictions on their movement into or out of these locations.

Given the reciprocal and dynamic influence between the organizational level of the internal food environment and its surroundings^1^, it is crucial to assess the types of food establishments located near HEIs. Moreover, it is important to determine the presence of food swamps, areas characterized by a high density of establishments selling calorie-dense, unhealthy foods^3^.

In this context, most young adults in college do not consume vegetables as frequently or in the quantities recommended by the World Health Organization^4^. National studies^5-7^ have consistently reported reduced consumption of fruits and vegetables among young adults, along with lower adherence to other markers of healthy eating compared to other age groups, coupled with a high intake of ultra-processed foods. Similar patterns are observed among Brazilian college students, who exhibit high consumption of fast foods, alcoholic beverages, sugary drinks, and sweets^8,9^.

Although national and international studies have characterized UFE and highlighted efforts to promote a healthy food environment within HEIs^2,10-12^, research focusing on the external environment surrounding these institutions remains limited. This study, therefore, aimed to analyze the retail food environment and identify the presence of food swamps in the surroundings of public and private HEIs in a Brazilian metropolis.

## METHODS

An ecological study was conducted in the municipality of Belo Horizonte, the capital of the state of Minas Gerais, which has an estimated population of 2,530,701 inhabitants^13^, a demographic density of 7,167.00 inhabitants/km^2^, and a Municipal Human Development Index (MHDI) of 0.810^14^. This MHDI score is classified as very high human development^15^.

The database of HEIs for the year 2019 was provided by the Secretariat of State of Education of Minas Gerais (*Secretaria do Estado de Educação de Minas Gerais* - SEE-MG) and contained records for 304 institutions. Data from extinct institutions, those without undergraduate courses, and institutions offering only distance learning (*ensino à distância* - EAD) or EAD centers were excluded. Subsequently, the addresses of the institutions were updated according to information on the institutions' websites, and three additional HEIs were included in the database. The study focused exclusively on campuses or units offering in-person education, and in the end, the sample included in the study consisted of 81 HEI units.

A total of 47 educational institutions were analyzed in this study, 15 of which had more than one unit or campus, resulting in 81 units evaluated, with an average of 1.7 campuses/units per HEI. It is important to note that the sample included both different institutions and multiple campuses of the same HEI, as the inclusion criterion for the study was the provision of in-person education. Given that this is an ecological study, where location plays a crucial role in influencing the results, each HEI and its campuses offering in-person education were considered distinct units.

Information regarding the administrative category and per capita income of the census sector in which the institutions were located was incorporated into the HEI database. To calculate per capita income, data on total income and total population from the most recent census were utilized^16^. The resulting per capita income values were then categorized into tertiles (in Brazilian Reais) based on data from all census sectors in Belo Horizonte: 1^st^ tertile (159.60 - 571.57), 2^nd^ tertile (572.15 - 1,191.18), and 3^rd^ tertile (1,193.82 - 8,388.16). For two census sectors, corresponding to two HEIs, per capita income could not be determined due to the unavailability of income data for those sectors; one of these HEIs was public, while the other was private.

Data on food retail establishments were obtained from a secondary data source provided by the Secretariat of Finance of the State of Minas Gerais (*Secretaria de Estado de Fazenda de Minas Gerais* - SEF/MG). The database included information on the addresses and the National Classification of Economic Activities (*Classificação Nacional de Atividades Econômicas* - CNAE) of food sales establishments^17^ for the year 2019.

The analysis included only establishments that sell food for immediate consumption, such as bars, restaurants, bakeries, snack bars, grocery stores, candy retailers, street vendors, supermarkets, and hypermarkets. This selection was based on the relevance of these establishments in providing ready-to-eat food, a key consideration for the university population and their academic routines. Grocery stores, supermarkets, and hypermarkets were included because these establishments often sell food for immediate consumption, particularly ultra-processed foods^18^.

According to the technical study conducted by the Interministerial Chamber of Food and Nutrition Security (*Câmara Interministerial de Segurança Alimentar e Nutricional -* CAISAN), food retail establishments are classified into three categories:

1. Of purchasing fresh foods, where the acquisition of fresh or minimally processed foods accounts for more than 50% of the total foods sold;
2. Establishments for purchasing ultra-processed foods, where the acquisition of ultra-processed foods accounts for more than 50% of the foods sold;
3. Mixed establishments, where there is a predominance of culinary preparations or processed foods, or where neither unprocessed/minimally processed foods nor ultra-processed foods predominates^18^.

The establishments included in this study belong to two of these categories: those purchasing ultra-processed products and mixed establishments.

The unit of analysis used in this study was the 500-meter buffer zone, defined around a geographic point representing the HEIs. This type of buffer considers the surrounding area from a specific point and measures the distance using streets and sidewalks^19^. The 500-meter distance was selected based on the characteristics of the university population, primarily composed of young adults, as it is considered a short distance for most adults^20^.

The presence of food swamps around HEIs was assessed by adapting the method proposed by Hager et al.^3^, previously used by Peres et al.^21^. In the study by Hager et al.^3^, the euclidean buffer was applied, and neighborhoods with four or more establishments selling unhealthy foods, specifically convenience stores and small grocery stores, within the buffer area were classified as food swamps.

In this study, several adaptations were made to the aforementioned method, including the use of the buffer network, which shows a high correlation and substantial comparability with the euclidean buffer^22^. Additionally, in contrast to the original method, grocery stores, candy stores, and snack bars were included in the calculation of food swamps, as these establishments share characteristics with American convenience stores, a category less common in the Brazilian food environment. Snack bars were specifically chosen to replace convenience stores, as they are frequently visited by the Brazilian population^23^ and represented a more prominent category in the database used. If the total number of these establishments within the buffer zone was greater than or equal to four, the area was classified as a food swamp.

Based on the location of the HEIs and the city's street network, the proximity measure was analyzed, specifically the distance (in meters) between the HEIs and food outlets, categorized by type. This analysis considered the walking routes that the university population would take, accounting for the connectivity of the streets to the nearest establishment.

ArcGIS - ArcMap Desktop version 10.8 software was used to develop the buffer networks and calculate the distances between the HEIs and the nearest establishments. Mapping, as well as the identification of HEIs and food establishments located within the buffer area, were performed using QGIS 3.22.6 software.

For the descriptive analysis, measures of frequency, central tendency (median), and dispersion (interquartile range - IQ) were evaluated. The medians of establishments selling food for immediate consumption within the buffer area and the medians of the distances between the HEIs and the nearest establishments were compared. Both analyses were conducted according to the administrative categories of the HEIs and the tertiles of *per capita* income in Belo Horizonte. The non-parametric Mann-Whitney test was applied in both cases. A significance level of p<0.05 (5%) was adopted for the interpretation of the results, and values below this threshold were considered statistically significant.

The frequencies of HEIs located in food swamps were also evaluated based on the tertiles of average *per capita* income in the municipality. The data obtained were analyzed using Stata software, version 14.0.

As the data used in this study are non-nominal public data, approval from a Research Ethics Committee was not required, in accordance with National Health Council Resolution No. 510/16, dated April 7, 2016.

## RESULTS

Among the 81 HEI units evaluated in the city of Belo Horizonte, 83.95% (n=68) were private institutions, and 16.05% (n=13) were public institutions. In both administrative categories, most HEIs were situated in higher-income census sectors, with 84.61% (n=11) of public institutions and 72.05% (n=49) of private institutions located in the 3^rd^ tertile of mean *per capita* income.

Regarding the characterization of the food environment, at least one establishment selling food for immediate consumption was present in 98.76% of the evaluated buffers. The predominant categories around HEIs were restaurants (98.76%), snack bars (97.53%), and street vendors (96.29%) (Table 1).

**Table 1:** Distribution of food establishments for immediate consumption around Higher Education Institutions, 500-meter buffer network. Belo Horizonte (MG), 2019.

Establishments	Higher Education Institutions with at least one establishment	
	n	%
Candy stores	57	70.37
Supermarkets and hypermarkets	62	76.54
Bakeries	67	82.71
Grocery stores	70	86.41
Bars	75	92.59
Street vendors	78	96.29
Snack bars	79	97.53
Restaurants	80	98.76

When assessing the density of establishments selling food in the surroundings of HEIs, the most frequent categories were snack bars (26; 12-49), restaurants (24; 11-47), and bars (8; 5-14), with the total median number of establishments being 73 (44-115) (Table 2). Regarding the frequency of establishments according to the administrative categories of HEIs, it was found that the median number of all food establishment categories was higher in the surroundings of private institutions compared to public HEIs. The Mann-Whitney test revealed statistically significant differences between the categories of street vendors (p=0.0009), bars (p=0.0074), snack bars (p=0.0049), and grocery stores (p=0.0161).

**Table 2:** Distribution of food establishments for immediate consumption around Higher Education Institutions in a 500-meter buffer network, by administrative category and income of the census sector. Belo Horizonte (MG), 2019.

Establishments	Total HEIs (n=81)	Administrative Category			Per Capita Income of Census Sector (R$)		
		Public (n=13)	Private (n=68)	p-value	1^st^ and 2^nd^ terciles (n=19)	3^rd^ tercile (n=60)	p-value
	Median (IQ)	Median (IQ)	Median (IQ)		Median (IQ)	Median (IQ)	
Supermarkets and hypermarkets	2 (1-3)	1 (0-2)	2 (1-3)	0.0611	2 (0-3)	2 (1-3)	0.5049
Candy stores	2 (0-4)	1 (0-3)	2 (0-4)	0.1810	1 (0-3)	2 (0-4)	0.2908
Bakeries	3 (1-5)	2 (0-4)	3 (1-5)	0.1475	2 (1-3)	3 (2-6)	0.0137
Grocery stores	3 (1-5)	1 (0-3)	3 (2-5)	0.0161	2 (2-3)	3 (1-5)	0.3812
Street vendors	4 (2-6)	1 (1-3)	4 (2-6)	0.0009	3 (1-5)	4 (2-6)	0.2772
Bars	8 (5-14)	5 (0-8)	8 (6-14)	0.0074	7 (4-7)	8 (6-14)	0.0384
Restaurants	24 (11-47)	17 (4-34)	26 (12-49.5)	0.0881	7 (5-17)	34 (18-52)	0.0000
Snack bars	26 (12-49)	15 (6-16)	28 (16-55.5)	0.0049	18 (7-36)	29.5 (16-58)	0.0305
Total	73 (44-115)	41 (16-67)	84.5 (49-132.5)	0.0104	52 (24-81)	92.5 (54.5-142)	0.0015

HEIs: higher education institutions; IQ: interquartile range.

When stratifying the HEIs by the mean *per capita* income of the census sector, a higher density of all categories of establishments selling food for immediate consumption was observed around HEIs located in higher-income areas (3^rd^ tertile), with the exception of supermarkets and hypermarkets. These establishments presented the same median values for both income categories in the 1^st^ and 2^nd^ tertiles (2; 0-3) and in the 3^rd^ tertile (2; 1-3). However, a significant difference was found for the categories of bars (p=0.0384), snack bars (p=0.0305), bakeries (p=0.0137), and restaurants (p<0.001) (Table 2).

It is worth noting that private HEIs located in the highest-income census sectors had a higher number of snack bars in their surroundings, as well as a greater total number of establishments selling food for immediate consumption within the buffer area. Finally, the median total number of establishments was significantly higher in the surroundings of private HEIs (p=0.0104), as well as in the surroundings of HEIs located in higher-income areas (p=0.0015) (Table 2).

**Figure 1:**
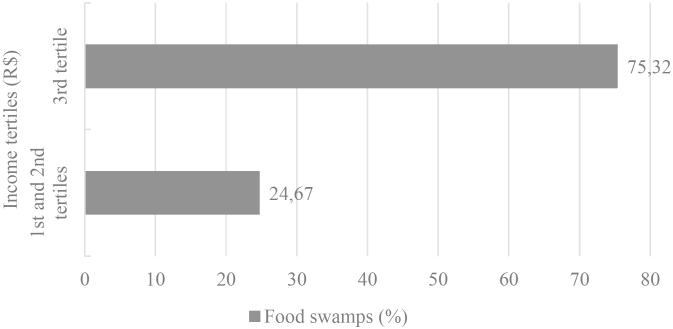
Frequency of food deserts in the surroundings of Higher Education Institutions, 500-meter buffer network. Belo Horizonte (MG), 2019.

Note: 1^st^ tertile (R$159.60 | R$571.57), 2^nd^ tertile (R$572.15 | R$1,191.18), and 3^rd^ tertile (R$1,193.82 | R$8,388.16).

Regarding establishments selling unhealthy foods, the results indicated that 95.06% (n=77) of the HEIs were located in neighborhoods identified as food swamps. Moreover, food swamps were more frequent around institutions situated in higher-income areas (3^rd^ tertile - 75.32%) (Figure 1).

When assessing the distance between HEIs and the nearest food retail establishments, it was observed that snack bars (47.5; 17.5-102.1 meters), restaurants (87.8; 30.9-150.8 meters), and bars (186.2; 105.7-308.1 meters) were the categories located at the shortest distances. In contrast, the category with the greatest distance was candy retail (381.0; 242.7-592.8 meters) (Table 3).

**Table 3:** Minimum distance, in meters, between Higher Education Institutions and food retail establishments for immediate consumption in the surrounding area, according to administrative category and income of the census sector. Belo Horizonte (MG), 2019.

Establishments	Administrative Category				*Per capita* income of census sector (R$)		
	Total HEIs* (n=80)	Public (n=12)	Private (n=68)	p-value†	1^st^ and 2^nd^ terciles (n=19)‡	3^rd^ tercile (n=59)‡	p-value†
	Median (IQ)	Median (IQ)	Median (IQ)		Median (IQ)	Median (IQ)	
Snack bars	47.5 (17.5-102.1)	28.3 (15.9-161.1)	52.3 (19.9-95.6)	0.627	54.6 (20.9-78.8)	42.8 (15.9-131.7)	0.925
Restaurants	87.8 (30.9-150.8)	49.60 (20.0-163.6)	92.4 (37.8-150.8)	0.627	65.2 (40.3-145.9)	91.3 (28.4-154.6)	0.788
Bars	186.2 (105.7-308.1)	235.6 (89.2-336.9)	178.1 (105.7-290.5)	0.434	190.3 (112.1-294.0)	182.2 (92.3-315.1)	0.492
Street vendors	273.5 (167.7-369.4)	274.4 (121.1-398.6)	273.5 (179.2-356.5)	0.903	272.2 (175.0-308.4)	276.1 (151.8-376.8)	0.666
Supermarkets and hypermarkets	280.5 (181.6-477.5)	210.5 (45.4-487.5)	286.2 (210.8-477.5)	0.305	272.1 (198.8-427.0)	289.3 (145.9-532.8)	0.953
Grocery stores	291.9 (149.3-392.0)	232.4 (142.4-344.7)	298.4 (154.7-401.5)	0.466	312.8 (173.1-386.3)	282.7 (133.6-407.4)	0.861
Bakeries	298.2 (163.6-411.0	311.9 (222.0-379.5)	298.2 (156.6-411.4)	0.492	286.6 (142.6-370.2)	308.9 (167.4-476.5)	0.389
Candy stores	381.0 (242.7-592.8)	370.7 (237.6-574.6)	381.0 (250.0-592.8)	0.850	303.1 (221.6-588.2)	402.9 (252.1-628.6)	0.522

HEIs: higher education institutions; IQ: interquartile range.

* 1 HEI did not obtain results for the analysis of the distance to the nearest establishment;

† p-values according to the Mann-Whitney test;

‡ For 02 HEIs, it was not possible to establish *per capita* income due to the absence of income data for the census sector.

The analysis of distances according to the administrative category of the HEIs showed that public HEIs were generally closer to the food establishments evaluated, except for street vendors, bars, and bakeries, which were closer to private HEIs. When evaluating distances based on the income of the sector, a greater proximity of establishments to HEIs located in lower-income areas was observed, except for bars, snack bars, and grocery stores, which were located closer to HEIs in higher-income areas. No significant differences were found between the distances for any category of establishments selling food for immediate consumption (Table 3).

## DISCUSSION

The findings of this study indicated a high density of establishments selling unhealthy food for immediate consumption in the surroundings of HEIs in the city of Belo Horizonte. This distribution was heterogeneous across the territory, varying according to the income level and administrative category of the institutions. Additionally, there was greater proximity of these categories of establishments to HEIs.

As in the national scenario, the predominant presence of private HEIs was observed (83.95%), a trend seen in 88.4% of Brazilian institutions^24^. This outcome is the result of a historical process, marked by numerous changes, including university reform. Over the past few decades, private HEIs have undergone a significant expansion, leading to the current disparity between the public and private sectors across the country^25^.

In addition to the quantitative disparity between public and private HEIs, the findings of this study also revealed differences in the food environment around them, with higher density of food establishments around private HEIs or in higher-income areas. Consistent with studies conducted in the same municipality^26,27^ and in other states in the country^28,29^, it was observed that an increase in income in the sector was accompanied by a rise in the density of all categories of establishments evaluated. This characteristic is proposed to result from the variety of activities in central areas of Brazilian municipalities, which are associated with better street connectivity^26^.

The findings related to snack bars are particularly noteworthy, as they not only had a frequent presence in the buffers evaluated but also exhibited the highest density and proximity to HEIs compared to other categories. According to national studies^18,23^, snack bars are among the most frequently visited establishments for purchasing food for immediate consumption. They are also characterized by the predominant sale of ultra-processed foods, such as snacks, soft drinks, and fast food.

It is well-established that the eating patterns of university students are inadequate, characterized by high consumption of snacks, fast food, sweets, cakes, pies, chips, and carbonated drinks, with a lower intake of fruits, vegetables, and whole grains^8^. In Brazil, data from the Risk and Protection Factors Surveillance System for Chronic Diseases through Telephone Survey (*Sistema de Vigilância de Fatores de Risco e Proteção para Doenças Crônicas por Inquérito Telefônico* - VIGITEL) further support these findings. The research shows that the highest consumption of soft drinks and ultra-processed food groups occurs in individuals aged 18 to 24 years. Similarly, this age group also has the lowest proportion of consumption of healthy eating markers, such as non- or minimally processed foods, as well as fruits and vegetables, compared to other age groups^7^.

In addition to the higher density of snack bars, over 90% of the buffers surrounding HEIs were classified as food swamps. Given the nutritional quality of ultra-processed foods, which are predominantly sold in these establishments, the surrounding food environment can be characterized as unhealthy. The prevalence of unhealthy food options in the vicinity can influence individuals' ability to form and maintain healthy eating behaviors^30^. Furthermore, it exposes the university population to a range of health risks, including noncommunicable diseases (NCDs), overweight, and obesity^31^.

Although studies on this topic are limited, a study conducted in the USA revealed a similar context, with an unhealthy food environment surrounding HEIs. The authors found that convenience stores were the most frequent type of establishment in the areas evaluated, followed by bakeries, pastry shops, candy stores, and ice cream parlors; supermarket chains; and alcoholic beverage^32^.

Although different methods were employed, the present study found similar characteristics in the food environment surrounding universities, with a higher density of all categories of unhealthy food retail establishments, regardless of the mode of transportation used^32^. Similarly, another study that assessed both UFE and surrounding establishments indicated the presence of healthier food options within the campus compared to those in the surrounding areas^33^.

When compared to other categories of food establishments, the closer proximity of cafeterias to HEIs highlights a context that may increase university students' vulnerability to the negative impacts of consuming ultra-processed foods and make it more difficult for them to adopt a healthy diet. The shorter distance and higher density of establishments selling unhealthy foods around the university environment were also emphasized in another study conducted in Belo Horizonte^21^.

The findings of this study also indicated that at least one bar was present in the surroundings of more than 90% of the HEIs, making it a frequent category in the buffers evaluated, regardless of the income or administrative category of the institution. Since bars predominantly offer unhealthy foods and beverages^18^, including ultra-processed foods and alcoholic beverages, their high availability and proximity to HEIs warrants caution. Both the consumption of ultra-processed foods (inadequate food consumption) and alcoholic beverages (excessive consumption) are considered risk factors NCDs^34^.

Data from national health surveillance studies indicate that both weekly alcohol consumption^5^ and excessive drinking^7^ are most prevalent in the age range of 18 to 29 years, which corresponds to the typical age of university students. Similarly, other studies have shown a higher prevalence of alcohol consumption among young university students and graduates of higher education institutions^35-38^. Ther fore, the significant presence of bars around HEIs may negatively impact the health of young university students by encouraging the consumption of alcoholic beverages.

Among the strengths of this study, its innovation is particularly noteworthy, as it is the first to evaluate the presence of food swamps around HEIs. This approach has been little explored in national studies on UFE. To date, most of the research conducted in Brazil that investigated the food environment around organizations has focused on the surroundings of schools^21,39-41^.

Another distinctive feature of this study is its evaluation of the surroundings of both public and private HEIs, as most Brazilian studies on the university food environment have prioritized the assessment of public HEIs^12,42,43^. Additionally, the use of the buffer network is a notable advantage, as it takes into account street connectivity, thereby providing results that are more aligned with the actual routes and distances students would need to cover.

A limitation of this study is that the category of street vendors only includes establishments that are registered with municipal control bodies. It is likely that the numbers presented for this category underestimate the actual quantity and distribution of street vendors, as many may operate informally and not be captured in official records.

Finally, although the focus of this study is on the food environment surrounding HEIs, it is believed that its results highlight the importance of implementing food and nutrition policies at the institutional level, in addition to research and extension projects on the topic. Ensuring a healthier internal food environment can discourage the search for food and meals in the surroundings of HEIs.

Due to the autonomy they have, HEIs can adjust contracts with food sales companies within their campuses to improve the food quality and nutritional value of the products sold in their scope. In the case of public HEIs, additional measures — such as expanding University Restaurants and promoting access to subsidized meals for the entire academic community — are important, as well as providing support to students through student retention policies related to food security and nutrition actions. As for the surroundings, this should be addressed through public policies, since the management of the institutions has no control over the external food context of their units.

## References

[1] Castro IRR, Canella DS (2022). Organizational food environments: advancing their conceptual model. Foods.

[2] Roy R, Soo D, Conroy D, Wall CR, Swinburn B (2019). Exploring university food environment and on-campus food purchasing behaviors, preferences, and opinions. J Nutr Educ Behav.

[3] Hager ER, Cockerham A, O'Reilly N, Harrington D, Harding J, Hurley KM (2017). Food swamps and food deserts in Baltimore city, MD, USA: associations with dietary behaviours among urban adolescent girls. Public Health Nutr.

[4] Rodrigues VM, Bray J, Fernandes AC, Bernardo GL, Hartwell H, Martinelli SS (2019). Vegetable consumption and factors associated with increased intake among college students: a scoping review of the last 10 years. Nutrients.

[5] Instituto Brasileiro de Geografia e Estatística (2020). Pesquisa nacional de saúde: 2019: percepção do estado de saúde, estilos de vida, doenças crônicas e saúde bucal: Brasil e grandes regiões [Internet].

[6] Brasil (2023). Inquérito telefônico de fatores de risco para doenças crônicas não transmissíveis em tempos de pandemia - Covitel 2: relatório final [Internet].

[7] Brasil. Ministério da Saúde. Secretaria de Vigilância em Saúde (2022). Departamento de Análise em Saúde e Vigilância de Doenças Não Transmissíveis. Vigitel Brasil 2021: vigilância de fatores de risco e proteção para doenças crônicas por inquérito telefônico: estimativas sobre frequência e distribuição sociodemográfica de fatores de risco e proteção para doenças crônicas nas capitais dos 26 estados brasileiros e no Distrito Federal em 2021 [Internet].

[8] Bernardo GL, Jomori MM, Fernandes AC, Proença RPC (2017). Food intake of university students. Rev Nutr.

[9] Barros GR, Santos SFSS, Andaki ACR, Sousa TF (2021). Sobrepeso e obesidade em universitários: prevalências e fatores associados. Rev Bras Ativ Fís Saúde.

[10] Pulz IS, Martins PA, Feldman C, Veiros MB (2017). Are campus food environments healthy? A novel perspective for qualitatively evaluating the nutritional quality of food sold at foodservice facilities at a Brazilian university. Perspect Public Health.

[11] Nogueira LR, Fontanelli MM, Aguiar BS, Failla MA, Florindo AA, Leme AC (2020). Is the local food environment associated with excess body weight in adolescents in São Paulo, Brazil?. Cad Saúde Pública.

[12] Barbosa R, Henriques P, Guerra H, Emerentino J, Soares D, Dias P (2020). Food environment of a Brazilian public university: challenges to promote healthy eating. Rev Chil Nutr.

[13] Instituto Brasileiro de Geografia e Estatística. Brasil (2021). Minas Gerais. Belo Horizonte. Panorama. População [Internet].

[14] Instituto Brasileiro de Geografia e Estatística (2011). Censo Demográfico 2010 [Internet].

[15] Programa das Nações Unidas para o Desenvolvimento (2013). Atlas do Desenvolvimento Humano no Brasil 2013.

[16] Instituto Brasileiro de Geografia e Estatística (2016). Metodologia do censo demográfico 2010.

[17] Instituto Brasileiro de Geografia e Estatística (2011). Classificação nacional de atividades econômicas [Internet].

[18] Câmara Interministerial de Segurança Alimentar e Nutricional (2018). Estudo Técnico - Mapeamento dos desertos alimentares no Brasil [Internet].

[19] Gamba RJ, Schuchter J, Rutt C, Seto EYW (2015). Measuring the food environment and its effects on obesity in the United States: a systematic review of methods and results. J Community Health.

[20] Walker BB, Shashank A, Gasevic D, Schuurman N, Poirier P, Teo K (2020). The local food environment and obesity: evidence from three cities. Obesity (Silver Spring).

[21] Peres CMC, Costa BVL, Pessoa MC, Honório OS, Carmo AS, Silva TPR (2021). O ambiente alimentar comunitário e a presença de pântanos alimentares no entorno das escolas de uma metrópole brasileira. Cad Saúde Pública.

[22] Burgoine T, Alvanides S, Lake AA (2013). Creating ‘obesogenic realities'; do our methodological choices make a difference when measuring the food environment?. Int J Health Geogr.

[23] Bezerra IN, Moreira TM, Cavalcante JB, Souza AM, Sichieri R (2017). Food consumed outside the home in Brazil according to places of purchase. Rev Saúde Pública.

[24] Brasil. Ministério da Educação (2021). Resumo técnico do Censo da Educação Superior 2019 [Internet].

[25] Broch C, Breschiliare FCT, Barbosa-Rinaldi IP (2020). A expansão da educação superior no Brasil: notas sobre os desafios do trabalho docente. Avaliação (Campinas).

[26] Lopes MS, Martiniano MO, Freitas PP, Carvalho MCR, Sales DM, Lopes ACS (2022). Comércio de alimentos para consumo imediato no entorno do Programa Academia da Saúde: uma análise segundo desigualdades. Ciênc Saúde Coletiva.

[27] Rocha LL, Carmo AS, Jardim MZ, Leme BA, Cardoso LO, Caiaffa WT (2023). The community food environment of a Brazilian metropolis. Food Cult Soc.

[28] Barbosa BB, Penha EDS, Carioca AAF (2022). Food environment of the economic capital of the Northeast: social and territorial disparities in the availability of food stores. Rev Nutr.

[29] Fortes MF, Borges CA, Miranda WC, Jaime PC (2018). Mapeando as desigualdades socioeconômicas na distribuição do comércio varejista local. Segur Aliment Nutr.

[30] Murray S, Peterson C, Primo C, Elliott C, Otlowski M, Auckland S (2021). Prevalence of food insecurity and satisfaction with on-campus food choices among Australian university students. Int J Sustain High Educ.

[31] Chen X, Zhang Z, Yang H, Qiu P, Wang H, Wang F (2020). Consumption of ultra-processed foods and health outcomes: a systematic review of epidemiological studies. Nutr J.

[32] Vilme H, Paul CJ, Duke NN, Campbell SD, Sauls D, Muiruri C (2022). Using geographic information systems to characterize food environments around historically black colleges and universities: Implications for nutrition interventions. J Am Coll Health.

[33] Horacek TM, Erdman MB, Byrd-Bredbenner C, Carey G, Colby SM, Greene GW (2013). Assessment of the dining environment on and near the campuses of fifteen post-secondary institutions. Public Health Nutr.

[34] World Health Organization (2018). Global status report on noncommunicable diseases 2018.

[35] Souza LPS, Hermsdorff HHM, Miranda AES, Bressan J, Pimenta AM (2021). Consumo de bebidas alcoólicas e excesso de peso em adultos brasileiros - Projeto CUME. Ciênc Saúde Coletiva.

[36] Fórum Nacional de Pró-Reitores de Assuntos Estudantis (2019). V Pesquisa Nacional de Perfil Socioeconômico e Cultural dos (as) Graduandos (as) das IFES - 2018 [Internet].

[37] Barros MSMR, Costa LS (2019). Perfil do consumo de álcool entre estudantes universitários. SMAD Rev Eletrônica Saúde Mental Álcool Drog.

[38] Dázio EMR, Zago MMF, Fava SMCL (2016). Uso de álcool e outras drogas entre universitários do sexo masculino e seus significados. Rev Esc Enferm USP.

[39] Andretti B, Cardoso LO, Honório OS, Castro PCPC, Tavares LF, Silva ICG (2023). Ecological study of the association between socioeconomic inequality and food deserts and swamps around schools in Rio de Janeiro, Brazil. BMC Public Health.

[40] Novaes TG, Mendes LL, Almeida LFF, Ribeiro AQ, Costa BVL, Claro RM (2022). Availability of food stores around Brazilian schools. Cien Saude Colet.

[41] Henriques P, Alvarenga CRT, Ferreira DM, Dias PC, Soares DSB, Barbosa RMS (2021). Ambiente alimentar do entorno de escolas públicas e privadas: oportunidade ou desafio para alimentação saudável?. Ciênc Saúde Coletiva.

[42] Sodré BE, Leite MA, Binoti ML (2021). Ambiente obesogênico universitário: achados de uma cidade brasileira de grande porte. R Assoc Bras Nutr.

[43] Franco AS, Canella DS, Perez PMP, Bandoni DH, Castro IRR (2020). Ambiente alimentar universitário: caracterização e mudanças no período de 2011 a 2016 em uma universidade pública brasileira. Rev Nutr.

