# Supplementary material for: Retail food environment around higher education institutions in a Brazilian metropolis
Source: Rev Bras Epidemiol. 2025 Jan 31;28:e250004. doi: 10.1590/1980-549720250004 (PMC11809084; doi:10.1590/1980-549720250004)
Supplement: Translation

# Translation: Ambiente alimentar de varejo no entorno de instituições de ensino superior de uma metrópole brasileira

**DOI:** 10.1590/1980-549720250004.2

**Authors:** Larissa Edwiges Ananda da Silva, Thales Philipe Rodrigues da Silva, Olívia Souza Honório, Monique Louise Cassimiro Inácio, Larissa Loures Mendes

**Affiliations:** I Universidade Federal de Minas Gerais, Programa de Pós-Graduação em Nutrição e Saúde - Belo Horizonte (MG), Brasil.; II Universidade Federal de Minas Gerais, Programa de Pós-Graduação em Enfermagem - Belo Horizonte (MG), Brasil.; III Universidade Federal de São Paulo, Escola Paulista de Enfermagem, Departamento de Enfermagem na Saúde da Mulher - São Paulo (SP), Brasil.; IV Universidade Federal de Ouro Preto, Programa de Pós-Graduação em Saúde e Nutrição - Ouro Preto (MG), Brasil.; V Universidade Federal de Minas Gerais, Departamento de Nutrição - Belo Horizonte (MG), Brasil.

**Keywords:** Ambiente construído, Universidades, Adulto jovem, Mapeamento geográfico, Saúde pública

## Abstract

**Objetivo:**

Analisar o ambiente alimentar de varejo e identificar a presença de pântanos alimentares no entorno de instituições de ensino superior (IES) públicas e privadas de Belo Horizonte, Minas Gerais.

**Métodos:**

Trata-se de estudo ecológico, que teve como unidade de análise o *buffer network* de 500 metros, traçado no entorno de 81 unidades de IES com ensino presencial. Foram avaliadas a densidade e a proximidade entre as IES e estabelecimentos de aquisição de alimentos para consumo imediato, de acordo com a categoria administrativa e renda *per capita* do setor censitário, além da presença de pântanos alimentares.

**Resultados:**

Em 98,76% dos *buffers* existia pelo menos um estabelecimento para consumo imediato. Lanchonetes, restaurantes e bares foram as categorias mais disponíveis e mais próximas das IES. A densidade de estabelecimentos foi maior no entorno das IES privadas e no entorno das IES localizadas em áreas de maior renda. Verificou-se que 95,06% das IES estavam localizadas em áreas classificadas como pântanos alimentares.

**Conclusão:**

Dessa forma, as IES avaliadas encontravam-se expostas a vizinhanças com ambiente alimentar não saudável, que podem predispor o público universitário a escolhas alimentares baseadas no consumo de alimentos ultraprocessados e bebidas alcoólicas.

## INTRODUÇÃO

O ambiente alimentar organizacional das universidades ou ambiente alimentar universitário (AAU), é caracterizado por elementos de seu ambiente físico interno, sendo proposto que o seu entorno, seja um dos seus componentes^1^. De acordo com Castro et al.^1^, o entorno refere-se aos contextos físicos e "virtuais" relacionados aos alimentos disponíveis para as pessoas que frequentam um determinado ambiente organizacional e que não são influenciados pela administração dessa organização. O contexto físico inclui estabelecimentos de venda de alimentos, bebidas e preparações culinárias, além de considerar também o comércio informal de tais produtos em áreas próximas à organização.

Sabe-se que os universitários costumam trazer refeições de casa ou comprar alimentos fora do *campus* ^2^, sendo assim, o impacto do AAU nos hábitos alimentares não se restringe ao que está disponível no interior das instituições de ensino superior (IES). Ademais, o público universitário, constituído predominantemente por jovens adultos, possui autonomia para adquirir alimentos no entorno da instituição de ensino, uma vez que não existe um controle de entrada ou saída desse público em tais locais.

Considerando a existência da influência recíproca e dinâmica entre o nível organizacional do ambiente alimentar interno e o entorno^1^, é fundamental avaliar os tipos de estabelecimentos comercializadores de alimentos presentes nas proximidades das IES. Além disso, deve-se verificar a existência dos pântanos alimentares, que são consideradas vizinhanças onde há grande concentração de estabelecimentos que comercializam alimentos altamente calóricos e não saudáveis^3^.

Neste contexto, os jovens adultos na universidade não consomem, em sua maioria, vegetais com a frequência recomendada pela Organização Mundial da Saúde, nem em quantidades suficientes^4^. Pesquisas nacionais^5-7^ evidenciam o consumo reduzido de frutas e hortaliças por jovens adultos, assim como de outros marcadores de alimentação saudável quando comparado aos demais grupos etários e, um elevado consumo de alimentos ultraprocessados. Entre os universitários brasileiros, os resultados são similares, com alto consumo de *fast foods*, bebidas alcoólicas, bebidas açucaradas e doces^8,9^.

Apesar da existência de pesquisas no âmbito nacional e internacional, que caracterizam o AAU e que apresentam esforços para promover um ambiente alimentar saudável no interior das IES^2,10-12^, ainda são escassos os estudos que se destinam a avaliar o ambiente do entorno de tais instituições. Assim, este estudo objetivou analisar o ambiente alimentar de varejo e identificar a presença de pântanos alimentares no entorno de IES públicas e privadas de uma metrópole brasileira.

## MÉTODOS

Estudo ecológico, realizado no município de Belo Horizonte, capital do estado de Minas Gerais, o qual possui população estimada de 2.530.701 habitantes^13^, densidade demográfica de 7.167,00 hab/km^2^ e Índice de Desenvolvimento Humano Municipal (IDHM) de 0,810^14^, sendo esse resultado classificado como desenvolvimento humano muito alto^15^.

O banco de dados das IES, referente ao ano de 2019, foi fornecido pela Secretaria do Estado de Educação de Minas Gerais (SEE-MG) e apresentava registros de 304 instituições. Foram excluídos os dados de IES extintas, sem cursos de graduação, além de IES com oferta exclusiva de ensino à distância (EAD), ou polos EAD. Posteriormente, os endereços das IES foram atualizados de acordo com o apresentado no site das instituições e realizada a inclusão de três IES no banco. O estudo contemplou apenas as unidades ou *campi* com a oferta de ensino presencial e, no final, a amostra incluída no estudo consistiu em 81 unidades de IES.

Neste estudo, foram analisadas 47 instituições de ensino ao todo, das quais 15 possuíam mais de uma unidade ou campus, resultando em 81 unidades avaliadas, com uma média de 1,7 campus/unidade por IES. Destaca-se que o total da amostra incluída compreendeu diferentes instituições e diferentes campi de uma mesma IES, uma vez que o critério para inclusão no estudo foi a oferta de ensino presencial. Assim, por se tratar de um estudo ecológico, no qual a localização possui influência nos resultados, todas as IES e seus *campi* com ensino presencial foram considerados como unidades distintas.

Ao banco de dados das IES, foram agregadas as informações sobre categoria administrativa e a renda *per capita* do setor censitário no qual as instituições estavam localizadas. Para o cálculo da renda *per capita* foram utilizadas as informações de renda total e de população total dos setores censitários do último censo^16^. Os resultados obtidos foram categorizados em tercis de renda *per capita* (em Reais), com base nos dados de todos os setores censitários de Belo Horizonte: 1° tercil (159,60 -| 571,57); 2° tercil (572,15-| 1.191,18); e 3° tercil (1.193,82 -| 8.388,16). Para dois setores censitários, correspondentes à duas IES, não foi possível estabelecer a renda *per capita* devido à ausência de dados de renda do setor, sendo uma dessas IES pública e outra privada.

Os dados sobre os estabelecimentos de varejo de alimentos foram obtidos de fonte de dados secundários, de acordo com as informações fornecidas pela Secretaria de Estado de Fazenda de Minas Gerais (SEF/MG). O banco de dados continha as informações do endereço e da Classificação Nacional de Atividades Econômicas (CNAE) dos estabelecimentos de venda de alimentos^17^, referentes ao ano de 2019.

Considerou-se apenas os dados de estabelecimentos de aquisição de alimentos para consumo imediato, tais como: bares, restaurantes, padarias, lanchonetes, mercearias, varejistas de doces, serviços ambulantes, supermercados e hipermercados. Tal escolha baseou-se no fato de que esses estabelecimentos oferecem alimentos prontos para o consumo, uma característica relevante para o contexto do público universitário e sua rotina acadêmica. As categorias de mercearias, supermercados e hipermercados foram incluídas no estudo pelo fato de que nesses estabelecimentos é possível adquirir alimentos para consumo imediato, principalmente alimentos ultraprocessados^18^.

Segundo o estudo técnico realizado pela Câmara Interministerial de Segurança Alimentar e Nutricional (CAISAN), existem três categorias para classificação desses estabelecimentos, dentre elas:

1. De aquisição de alimentos *in natura,* onde a aquisição de alimentos *in natura* ou minimamente processados representa mais de 50% total de alimentos comercializados;
2. Estabelecimentos de aquisição de ultraprocessados, onde a aquisição de alimentos ultraprocessados representa mais de 50% dos alimentos comercializados;
3. Estabelecimentos mistos, nos quais há predominância de aquisição de preparações culinárias ou alimentos processados ou onde não há predominância de aquisição de alimentos *in natura*/minimamente processados nem de alimentos ultraprocessados^18^.

Os estabelecimentos incluídos neste estudo pertencem a duas dessas categorias, estabelecimentos de aquisição de ultraprocessados e estabelecimentos mistos.

A unidade de análise utilizada foi o *buffer network* de 500 metros, traçado a partir de um ponto geográfico representado pelas IES. Esse tipo de *buffer* considera a área geográfica a partir de um ponto específico, e mede a distância usando ruas e calçadas^19^. A distância adotada foi estabelecida segundo as características da população universitária, composta em sua maioria por jovens adultos, uma vez que um *buffer* de 500 metros é considerado uma distância curta para a maioria dos adultos^20^.

A presença de pântanos alimentares no entorno das IES foi avaliada por meio da adaptação do método proposto por Hager et al.^3^, já previamente utilizado por Peres et al.^21^. No estudo de Hager et al.^3^ foi utilizado o *buffer* euclidiano e considerados como pântanos alimentares as vizinhanças com quatro ou mais estabelecimentos de vendas de alimentos não saudáveis, no caso, as lojas de conveniência e pequenas mercearias, presentes na área do *buffer*.

No presente estudo foram realizadas adaptações ao método citado, como o emprego do *buffer network*, o qual possui uma elevada correlação e substancial comparabilidade com o *buffer* euclidiano^22^. Ademais, além das mercearias, para o cálculo dos pântanos considerou-se também as lojas varejistas de doces e lanchonetes, por serem estabelecimentos com características mais semelhantes às lojas de conveniência americanas, já que esta categoria não é tão presente no ambiente alimentar brasileiro. Ainda, as lanchonetes foram empregadas em substituição às lojas de conveniência, uma vez que compõem uma categoria de estabelecimentos mais frequentados pela população brasileira^23^, além de ser uma categoria mais frequente na base de dados utilizada. Quando o somatório desses estabelecimentos presentes no *buffer* for maior ou igual a quatro, a área avaliada é classificada como um pântano alimentar.

A partir da localização das IES e da malha de ruas da cidade, procedeu-se a análise da medida de proximidade, ou seja, a distância (em metros) entre as IES e os pontos de vendas de alimentos, por categorias. Essa análise considerou os trajetos a serem percorridos a pé pelo público universitário, conforme a conectividade das ruas, até o estabelecimento mais próximo.

O *software* ArcGIS - ArcMap Desktop versão 10.8 foi utilizado para a elaboração dos *buffers network* e cálculos das distâncias entre as IES e os estabelecimentos mais próximos. O mapeamento, assim como a contabilização das IES e dos estabelecimentos de vendas de alimentos localizados na área do *buffer* foram realizados com o auxílio do *software* QGis 3.22.6.

Para a análise descritiva, foram avaliadas as medidas de frequência, tendência central (mediana) e medida de dispersão (intervalo interquartil - IQ). Foram comparadas as medianas de estabelecimentos de vendas de alimentos para consumo imediato presentes na área dos *buffers* e as medianas das distâncias entre as IES e os estabelecimentos mais próximos. Ambas as análises foram realizadas de acordo com as categorias administrativas das IES e os tercis de renda *per capita* de Belo Horizonte e, nos dois casos, foi utilizado o teste não-paramétrico de Mann-Whitney. O nível de significância de p<0,05 (5%) foi adotado nos procedimentos analíticos para a interpretação dos resultados obtidos e considerado como estatisticamente significante.

Também foram avaliadas as frequências de IES localizadas em pântanos alimentares, de acordo com os tercis de renda média per capita do município. Os dados obtidos foram analisados no *software* Stata versão 14.0.

Por se tratar de dados públicos não nominais, não foi necessária a aprovação do projeto de estudo por um Comitê de Ética em Pesquisa, com base na Resolução do Conselho Nacional de Saúde no 510/16, de 07 de abril de 2016.

## RESULTADOS

Entre as 81 unidades de IES avaliadas no município de Belo Horizonte, 83,95% (n=68) eram instituições privadas e 16,05% (n=13) instituições públicas. Em ambas as categorias administrativas, as IES estavam localizadas, em sua maior parte, em setores censitários de maior renda, com 84,61% (n=11) das instituições públicas e 72,05% (n=49) das instituições privadas situadas no 3° tercil de renda média *per capita*.

Com relação à caracterização do ambiente alimentar, em 98,76% dos *buffers* avaliados existiam pelo menos um estabelecimento com venda de alimentos para consumo imediato, sendo predominante a presença das categorias de restaurantes (98,76%), lanchonetes (97,53%) e ambulantes (96,29%) no entorno das IES (Tabela 1).

**Tabela 1:** Distribuição dos estabelecimentos de venda de alimentos para consumo imediato no entorno de Instituições de Ensino Superior, *buffer network* de 500 metros. Belo Horizonte (MG), 2019.

Estabelecimentos	Instituições de Ensino Superior com pelo menos um estabelecimento	
	n	%
Varejistas de doces	57	70,37
Supermercados e hipermercados	62	76,54
Padarias	67	82,71
Mercearias	70	86,41
Bares	75	92,59
Ambulantes	78	96,29
Lanchonetes	79	97,53
Restaurantes	80	98,76

Quando avaliada a densidade de estabelecimentos que comercializavam alimentos no entorno das IES, verificou-se que as categorias mais frequentes foram as lanchonetes (26; 12-49), restaurantes (24; 11-47), e bares (8; 5-14), sendo a mediana total de estabelecimentos igual a 73 (44-115) (Tabela 2). Em relação à frequência de estabelecimentos de acordo com a categorias administrativas das IES, foi possível verificar que no entorno das instituições privadas, a mediana de todas as categorias de estabelecimentos de venda de alimentos foi maior, quando comparada às IES públicas. O Teste de Mann-Whitney apontou que essa diferença foi estatisticamente significante entre as categorias de ambulantes (p=0,0009), bares (p=0,0074), lanchonetes (p=0,0049) e mercearias (p=0,0161).

**Tabela 2:** Distribuição dos estabelecimentos de venda de alimentos para consumo imediato no entorno de Instituições de Ensino Superior em *buffer network* de 500 metros, segundo categoria administrativa e renda do setor censitário. Belo Horizonte (MG), 2019.

Estabelecimentos	Total IES (n=81)	Categoria administrativa			Renda per capita do setor censitário (R$)		
		Públicas (n=13)	Privadas (n=68)	p-valor	1º e 2º tercil (n=19)	3º tercil (n=60)	p-valor
	Mediana (IQ)	Mediana (IQ)	Mediana (IQ)		Mediana (IQ)	Mediana (IQ)	
Supermercados e Hipermercados	2 (1-3)	1 (0-2)	2 (1-3)	0,0611	2 (0-3)	2 (1-3)	0,5049
Varejistas de doces	2 (0-4)	1 (0-3)	2 (0-4)	0,1810	1 (0-3)	2 (0-4)	0,2908
Padarias	3 (1-5)	2 (0-4)	3 (1-5)	0,1475	2 (1-3)	3 (2-6)	0,0137
Mercearias	3 (1-5)	1 (0-3)	3 (2-5)	0,0161	2 (2-3)	3 (1-5)	0,3812
Ambulantes	4 (2-6)	1 (1-3)	4 (2-6)	0,0009	3 (1-5)	4 (2-6)	0,2772
Bares	8 (5-14)	5 (0-8)	8 (6-14)	0,0074	7 (4-7)	8 (6-14)	0,0384
Restaurantes	24 (11-47)	17 (4-34)	26 (12-49,5)	0,0881	7 (5-17)	34 (18-52)	0,0000
Lanchonetes	26 (12-49)	15 (6-16)	28 (16-55,5)	0,0049	18 (7-36)	29,5 (16-58)	0,0305
Total	73 (44-115)	41 (16-67)	84,5 (49-132,5)	0,0104	52 (24-81)	92,5 (54,5-142)	0,0015

IES: instituições de ensino superior; IQ: intervalo interquartil.

Ao estratificar as IES pela renda média *per capita* do setor censitário, observou-se uma maior densidade de todas as categorias de estabelecimentos de venda de alimentos para consumo imediato no entorno das IES localizadas em áreas de maior renda (3º tercil), exceto supermercados e hipermercados, que apresentaram os mesmos valores de mediana para ambas as categorias de tercis de renda avaliadas no 1° e 2° tercil (2; 0-3) e no 3º tercil (2; 1-3). Entretanto, foi observada uma diferença significativa para as categorias de bares (p=0,0384), lanchonetes (p=0,0305), padarias (p=0,0137) e restaurantes (p<0,001) (Tabela 2).

Ressalta-se que IES privadas, localizadas nos setores censitários de maior renda, apresentaram um número maior de lanchonetes em seu entorno, assim como o total de estabelecimentos para consumo imediato presentes na área do *buffer*. Por fim, a mediana do total de estabelecimentos foi significativamente maior no entorno de IES privadas (p=0,0104), assim como no entorno de IES localizadas em áreas de maior renda (p=0,0015) (Tabela 2).

Com relação aos estabelecimentos de venda de alimentos não saudáveis, os resultados apontaram que 95,06% (n=77) das IES estavam localizadas em vizinhanças denominadas como pântanos alimentares. Além disso, os pântanos foram mais frequentes no entorno das instituições que estavam localizadas em áreas de maior renda (3º tercil - 75,32%) (Figura 1).

**Figura 1:** Frequência de pântanos alimentares no entorno de Instituições de Ensino Superior, buffer network de 500 metros. Belo Horizonte (MG), 2019.

Nota: 1º tercil (R$159,60 -| R$571,57), 2º tercil (R$572,15-| R$1.191,18) e 3º tercil (R$1.193,82 -| R$8.388,16).

Ao avaliar a distância das IES aos estabelecimentos comercializadores de alimentos mais próximos, observou-se que as lanchonetes (47,5; 17,5-102,1 metros), restaurantes (87,8; 30,9-150,8 metros) e bares (186,2; 105,73-308,1 metros) foram as categorias localizadas a uma menor distância. Por outro lado, a categoria que apresentou a maior distância foi a varejista de doces (381,0; 242,7-592,8 metros) (Tabela 3).

**Tabela 3:** Distância mínima, em metros, entre as Instituições de Ensino Superior e estabelecimentos de venda de alimentos para consumo imediato do entorno, segundo a categoria administrativa e renda do setor censitário. Belo Horizonte (MG), 2019.

Estabelecimentos	Categoria administrativa				Renda per capita do setor censitário (R$)		
	Total IES* (n=80)	Públicas (n=12)	Privadas (n=68)	p-valor†	1º e 2º tercil (n=19)‡	3º tercil (n=59)‡	p-valor†
	Mediana (IQ)	Mediana (IQ)	Mediana (IQ)		Mediana (IQ)	Mediana (IQ)	
Lanchonetes	47,5 (17,5-102,1)	28,3 (15,9-161,1)	52,3 (19,9-95,6)	0,627	54,6 (20,9-78,8)	42,8 (15,9-131,7)	0,925
Restaurantes	87,8 (30,9-150,8)	49,60 (20,0-163,6)	92,4 (37,8-150,8)	0,627	65,2 (40,3-145,9)	91,3 (28,4-154,6)	0,788
Bares	186,2 (105,7-308,1)	235,6 (89,2-336,9)	178,1 (105,7-290,5)	0,434	190,3 (112,1-294,0)	182,2 (92,3-315,1)	0,492
Ambulantes	273,5 (167,7-369,4)	274,4 (121,1-398,6)	273,5 (179,2-356,5)	0,903	272,2 (175,0-308,4)	276,1 (151,8-376,8)	0,666
Supermercados e Hipermercados	280,5 (181,6-477,5)	210,5 (45,4-487,5)	286,2 (210,8-477,5)	0,305	272,1 (198,8-427,0)	289,3 (145,9-532,8)	0,953
Mercearias	291,9 (149,3-392,0)	232,4 (142,4-344,7)	298,4 (154,7-401,5)	0,466	312,8 (173,1-386,3)	282,7 (133,6-407,4)	0,861
Padarias	298,2 (163,6-411,0	311,9 (222,0-379,5)	298,2 (156,6-411,4)	0,492	286,6 (142,6-370,2)	308,9 (167,4-476,5)	0,389
Varejistas de doces	381,0 (242,7-592,8)	370,7 (237,6-574,6)	381,0 (250,0-592,8)	0,850	303,1 (221,6-588,2)	402,9 (252,1-628,6)	0,522

IES: instituições de ensino superior; IQ: intervalo interquartil.

* 1 IES não obteve resultados para análise da distância ao estabelecimento mais próximo;

† Valores de p segundo teste de Mann-Whitney;

‡ Para 02 IES não foi possível estabelecer a renda *per capita*, devido à ausência de dados de renda do setor censitário.

A análise das distâncias de acordo com a categoria administrativa das IES indico uma maior proximidade das IES públicas para as categorias de estabelecimentos avaliados, exceto para as categorias de ambulantes, bares e padarias, as quais apresentaram maior proximidade das IES privadas. No caso da avaliação das distâncias de acordo com a renda do setor, verificou-se uma maior proximidade dos estabelecimentos para IES situadas em áreas de menor renda, exceto para as categorias de bares, lanchonetes e mercearias, que indicaram menores distâncias até as IES em áreas de maior renda. Não foram observadas diferenças significativas entre as distâncias avaliadas para nenhuma categoria de estabelecimentos de venda de alimentos para consumo imediato (Tabela 3).

## DISCUSSÃO

Os achados deste estudo indicaram elevada densidade de estabelecimentos com comercialização de alimentos para consumo imediato não saudáveis no entorno das IES no município de Belo Horizonte, sendo que essa distribuição ocorreu de forma heterogênea no território, de acordo com a renda e categoria administrativa da instituição. Da mesma forma, verificou-se uma maior proximidade de tais categorias de estabelecimentos em relação às IES.

Assim como no cenário nacional, foi observada a presença predominante de IES privadas (83,95%), sendo essa categoria administrativa presente em 88,4% das instituições brasileiras^24^. Resultado de um processo histórico — no qual foram realizadas inúmeras modificações —, além da reforma universitária, as IES privadas tiveram um intenso processo de expansão nas últimas décadas, que culminou na atual discrepância entre setor público e privado em todo o país^25^.

Além da disparidade quantitativa entre IES públicas e privadas, os achados desse estudo indicaram também diferenças no ambiente alimentar do entorno delas, com maior densidade para todas as categorias de estabelecimentos localizados em vizinhanças de IES privadas ou em áreas de maior renda. Assim como estudos realizados no mesmo município^26,27^ e em outros estados do país^28,29^, observou-se que o aumento da renda do setor foi acompanhado pelo aumento na densidade para todas as categorias de estabelecimentos avaliados. Propõe-se que essa característica decorra de uma variedade de atividades em áreas centrais dos municípios brasileiros, associada à melhor conectividade de ruas^26^.

Destaca-se os achados relacionados às lanchonetes, uma vez que além de presença frequente nos *buffers* avaliados, foi aquela com a maior densidade e proximidade às IES quando comparada às demais. De acordo com estudos nacionais^18,23^, as lanchonetes são consideradas uma das categorias com maior frequência de aquisição de alimentos para consumo imediato, além de ser caracterizada pela venda predominante de alimentos ultraprocessados, como salgadinhos, refrigerantes e alimentos do tipo *fast food*.

Sabe-se que o padrão alimentar adotado por estudantes universitários é inadequado, caracterizado pela alta ingestão de lanches, *fast food*, doces, bolos, tortas, batatas fritas e bebidas gaseificadas, e uma menor ingestão de frutas, vegetais e grãos integrais^8^. No Brasil, os dados do Sistema de Vigilância de Fatores de Risco e Proteção para Doenças Crônicas por Inquérito Telefônico (VIGITEL) reforçam os achados, já que a pesquisa aponta que o maior consumo de refrigerantes e grupos de alimentos ultraprocessados ocorre na faixa etária dos indivíduos de 18 a 24 anos. Igualmente, o mesmo grupo apresenta a menor proporção de consumo para marcadores de alimentação saudável, como alimentos não ou minimamente processados, além de frutas e hortaliças, dentre todas as idades avaliadas^7^.

Além da maior densidade de lanchonetes, destaca-se a presença expressiva, superior a 90%, de *buffers* classificados como pântanos alimentares nos entornos das IES. Devido à qualidade nutricional dos alimentos ultraprocessados, predominantemente comercializados nesses estabelecimentos, o ambiente alimentar do entorno pode ser caracterizado como não saudável. Ao propiciar oportunidades para comercialização de alimentos não saudáveis, as características do ambiente do entorno podem afetar a capacidade de formar e manter comportamentos alimentares saudáveis^30^, além de expor o público universitário a diferentes desfechos em saúde, como doenças crônicas não transmissíveis (DCNT), excesso de peso e obesidade^31^.

Embora haja uma escassez de estudos sobre a temática, uma pesquisa realizada nos EUA indicou um contexto semelhante, com o ambiente alimentar pouco saudável no entorno das IES. Os autores verificaram que as lojas de conveniência representaram a categoria de estabelecimentos mais frequentes nas áreas avaliadas, seguidas por padarias, pastelarias, docerias e sorveterias; cadeia de supermercados; e lojas de bebidas alcoólicas^32^.

Ainda que tenha empregado métodos diferentes, o presente estudo encontrou características semelhantes no ambiente alimentar das vizinhanças das universidades, com maior densidade para todas categorias de estabelecimentos de varejo de alimentos não saudáveis, independentemente do tipo de deslocamento empregado^32^. Da mesma forma, outro estudo, que avaliou o AAU e estabelecimentos do entorno, indicou a presença de opções de alimentos mais saudáveis dentro do *campus* do que entre os estabelecimentos do entorno^33^.

Quando comparada às demais categorias de estabelecimentos que comercializam alimentos, a maior proximidade entre as IES e as lanchonetes evidencia um contexto que pode tornar o público universitário mais vulnerável aos impactos negativos do consumo de alimentos ultraprocessados e dificultar a adoção de uma alimentação saudável. A menor distância e a maior densidade de estabelecimentos que comercializam alimentos não saudáveis no entorno escolar também foram apontadas em outra pesquisa realizado em Belo Horizonte^21^.

Os achados deste estudo apontaram ainda a presença de pelo menos um bar no entorno de mais de 90% das IES, além de ser uma categoria frequente nos *buffers* avaliados, independente da renda ou categoria administrativa da instituição de ensino superior. Por se tratar de um tipo de estabelecimento com oferta predominante de alimentos e bebidas não saudáveis^18^, como ultraprocessados e bebidas alcoólicas, sua elevada disponibilidade e proximidade das IES requer cautela, visto que tanto o consumo de alimentos ultraprocessados (consumo alimentar inadequado) como de bebidas alcoólicas (consumo excessivo) são considerados como fatores de risco para as DCNT^34^.

Dados de estudos nacionais de vigilância em saúde indicaram que tanto o consumo de bebida alcoólica uma vez ou mais por semana^5^ quanto o consumo abusivo^7^ ocorrem na faixa de idade compreendida entre 18 e 29 anos, na qual inserem-se a maior parte dos universitários. Da mesma forma, outras pesquisas apontam uma maior prevalência de consumo de bebidas alcoólicas por parte de jovens universitários e egressos de IES^35-38^. Portanto, a expressiva disponibilidade de bares no entorno das IES pode impactar negativamente na saúde dos jovens universitários, ao favorecer o consumo de bebidas alcoólicas.

Dentre os pontos fortes do presente estudo, destaca-se a sua inovação por ser o primeiro a avaliar a presença de pântanos alimentares no entorno de IES, já que essa é uma abordagem pouco explorada nos estudos nacionais sobre o AAU. Até o momento, a maior parte dos estudos realizados no Brasil que investigaram o ambiente alimentar do entorno de organizações foram desenvolvidos em vizinhanças do ambiente escolar^21,39-41^.

Outra característica particular é o fato de avaliar o entorno de IES públicas e privadas, visto que, os estudos brasileiros relativos ao ambiente alimentar universitário priorizaram a avaliação de IES públicas^12,42,43^. Além disso, o emprego do *buffer network* pode ser considerado um diferencial, pois considera a conectividade de ruas e, portanto, produz resultados mais condizentes com as rotas e distâncias a serem percorridas.

Como limitações, deve-se ressaltar que a categoria de ambulantes utilizada nesta pesquisa considera apenas os estabelecimentos registrados nos órgãos de controle do município. Acredita-se que os números apresentados para essa categoria de atividade econômica estejam subestimados em relação ao seu real quantitativo e distribuição.

Por fim, apesar do foco deste estudo ser o ambiente alimentar do entorno das IES, considera-se que seus resultados evidenciam a importância de se implementar políticas de alimentação e nutrição à nível institucional, além de projetos de pesquisa e extensão sobre o tema. Garantir um ambiente alimentar interno mais saudável pode desestimular a busca de alimentos e refeições no entorno das IES.

Em virtude da autonomia que dispõem, as IES podem adequar os contratos com empresas de vendas de alimentos dentro dos *campi*, a fim de melhorar a qualidade alimentar e nutricional dos alimentos comercializados em sua área de abrangência. No caso das IES públicas, medidas adicionais — como ampliação dos Restaurantes Universitários e promoção do acesso a refeições subsidiadas a toda comunidade acadêmica — são importantes, assim como o atendimento aos universitários, por meio das políticas de permanência estudantil relacionadas as ações de segurança alimentar e nutricional. Quanto ao entorno, este deve ser trabalhado por meio de políticas públicas, já que a gestão das instituições não possui controle sobre o contexto alimentar externo de suas unidades.
